# Persistently high impact of alcohol use on fatal violence in Lithuania despite strengthening alcohol control policies, 2004–19

**DOI:** 10.1093/eurpub/ckaf083

**Published:** 2025-06-10

**Authors:** Domantas Jasilionis, Laura Miščikienė, Shannon Lange, Huan Jiang, Daumantas Stumbrys, Olga Meščeriakova, Mindaugas Štelemėkas, Jürgen Rehm

**Affiliations:** Laboratory of Demographic Data, Max Planck Institute for Demographic Research, Rostock, Germany; Vytautas Kavolis Transdisciplinary Research Institute, Vytautas Magnus University, Kaunas, Lithuania; Health Research Institute, Faculty of Public Health, Lithuanian University of Health Sciences, Kaunas, Lithuania; Institute for Mental Health Policy Research, Centre for Addiction and Mental Health, Toronto, ON, Canada; Campbell Family Mental Health Research Institute, Centre for Addiction and Mental Health, Toronto, ON, Canada; Department of Psychiatry, University of Toronto, Toronto, ON, Canada; Institute of Medical Science, University of Toronto, Toronto, ON, Canada; Institute for Mental Health Policy Research, Centre for Addiction and Mental Health, Toronto, ON, Canada; Dalla Lana School of Public Health, University of Toronto, Toronto, ON, Canada; Vytautas Kavolis Transdisciplinary Research Institute, Vytautas Magnus University, Kaunas, Lithuania; Health Research Institute, Faculty of Public Health, Lithuanian University of Health Sciences, Kaunas, Lithuania; Department of Health Management, Faculty of Public Health, Lithuanian University of Health Sciences, Kaunas, Lithuania; Health Research Institute, Faculty of Public Health, Lithuanian University of Health Sciences, Kaunas, Lithuania; Department of Preventive Medicine, Faculty of Public Health, Lithuanian University of Health Sciences, Kaunas, Lithuania; Institute for Mental Health Policy Research, Centre for Addiction and Mental Health, Toronto, ON, Canada; Campbell Family Mental Health Research Institute, Centre for Addiction and Mental Health, Toronto, ON, Canada; Department of Psychiatry, University of Toronto, Toronto, ON, Canada; Institute of Medical Science, University of Toronto, Toronto, ON, Canada; Dalla Lana School of Public Health, University of Toronto, Toronto, ON, Canada; Program on Substance Abuse & WHO European Region Collaboration Centre, Public Health Agency of Catalonia, Barcelona, Spain; Zentrum für Interdisziplinäre Suchtforschung (ZIS), Universitätsklinikum Hamburg-Eppendorf, Hamburg, Germany

## Abstract

A strong association between alcohol and violence and homicide has been well established. Much less is known about the relationship between alcohol policies and the perpetration of alcohol-involved homicides, especially in the Central and Eastern European region. Despite recent progress, Lithuania still has one of the highest alcohol per capita consumption and homicide rates in the European region. Using quarterly data on homicide perpetrators in Lithuania for 2004–19, interrupted time-series were performed to evaluate whether the 2017 and 2018 alcohol control policies had an impact on the rate of perpetrators of homicide and the proportion of perpetrators under the influence of alcohol using a generalized additive model and generalized linear model, respectively. Although a rapid decline was observed in both the absolute numbers of homicides and rates of homicide perpetrators between 2004 and 2019, the proportion of homicide perpetrators under the influence of alcohol remained high. The analyses revealed that there was no significant effect of either of the two alcohol control policies on the rate of homicide perpetrators or the proportion of perpetrators under the influence of alcohol. The problem of persistently high occurrence of alcohol-involvement in homicides cannot be addressed by implementing alcohol control policies alone and thus, requires more inter-sectorial policy actions. More research is needed to understand homicide contexts and factors from both the victim and perpetrator perspectives.

## Introduction

Prior evidence has consistently suggested a robust positive association between alcohol use and violence. Studies using various designs and methods have consistently reported that alcohol consumption is a common determinant for violent incidents, committing violent acts, and victimization in this context [1–3]. Alcohol use is closely related to the worst cases of violence—homicides. There is an extensive literature on levels of alcohol consumption and homicides [4–8]. For example, Latin American countries with higher alcohol consumption levels and more prevalent risky alcohol consumption patterns such as binge drinking maintain higher homicide rates than countries from the same continent with less harmful consumption patterns [9]. The same relationship was also found in the Central and Eastern European region [5, 10].

Despite there being solid existing evidence about alcohol involvement in homicides, relatively little is known about the relationship between alcohol control policies and alcohol-involved homicides, especially in the Central and Eastern European region. Trangenstein *et al.* [11] found that countries with policies that reduce alcohol’s affordability or days/hours of sales tend to have fewer alcohol-attributable homicides, regardless of their income level. Among the countries of the former USSR, the most consistent and well-documented policy effect on violent deaths is Gorbachev’s anti-alcohol campaign, leading to a remarkable decrease in alcohol-related violent deaths, including homicides [12, 13].

Despite recent progress, Lithuania still has one of the highest alcohol per capita consumption in the European region. The latest World Health Organization estimate for Lithuania for 2017–19 was 11.8 liters of absolute alcohol per adult (15+ years of age) [14]. Statistics Lithuania provides a more recent figure of 11.0 liters of recorded alcohol per adult over 15 years of age in 2023 [15]. At the same time, despite reaching the lowest level since 1981, the age-standardized homicide rate (2.1 per 100 000 population) in 2019 remained much higher than in Western European countries or even compared to Central European countries such as Poland (0.6 per 100 000 population) [16]. Prior research on Lithuania highlighted the important role of excessive alcohol consumption in explaining high mortality due to external causes of death. For example, a prior autopsy-based study found that over half (57%) of victims dying from external causes of death during 1985–2014 had alcohol in blood [17]. More recent autopsy data on victims for 2020 suggests an even a higher figure (63.2%) [18]. Two Lithuanian studies confirm that the majority of both victims and perpetrators were under the influence of alcohol [19, 20]. Several recent studies also found significant associations between the implementation of alcohol control policies and declining mortality due to traffic accidents and suicide [21, 22]. Yet, such associations have never explored the potential role of alcohol control policies in reducing alcohol involvement in homicide perpetrators. To our knowledge, the vast majority of international findings on the relationship between alcohol control policies and violence rely on the victim’s perspective. Using time series quarterly data on homicide perpetrators in Lithuania for 2004–19, this study aimed to explore:

- The trends in annual homicide rates and quarterly rates of homicide perpetrators over time;
- whether there were any significant changes in the proportion of perpetrators under the influence of alcohol; and
- whether the 2017 and 2018 alcohol control policies had an impact on the rate of homicide perpetrators and the proportion of perpetrators under the influence of alcohol.

## Methods

The dataset on quarterly perpetrator numbers and their characteristics (including information about detected alcohol in the blood) comes from the dataset provided by the Information Technology and Communications Department (ITCD) under the Ministry of the Interior of the Republic of Lithuania. In Lithuania, data on all the crimes committed/under investigation are collected and processed by the Criminal Offenses Official Register operating at the ITCD. The data provided have been restricted to the officially registered crimes with known exact dates and suspects (perpetrators) under a criminal investigation. The data on homicides refer to paragraphs 129 and 130 of the Criminal Code of the Republic of Lithuania, corresponding to code 0101 of the International Classification of Crime for Statistical Purposes code 0101 (intentional homicide). The data on each recorded crime include the information on the date of crime, the date of starting criminal investigation (assigning the status of a suspect), and the assigned article of criminal code. The data also include the major characteristics of each suspect assigned to the recorded crime, including age, sex, education, professional background, urban–rural place of residence, and municipality. In addition, all records also included information on substance use (including alcohol) for perpetrators at the time the crime was committed, according to the police records. The final dataset includes 64 time points (quarters) covering January 2004—December 2019.

In order to investigate the potential impact of alcohol control policies, we used two characteristics as dependent variables: (i) quarterly rates of homicide perpetrators per 100 000 population and (ii) quarterly proportions of perpetrators under the influence of alcohol from the total number of perpetrators. Following the Interrupted Time Series (ITS) requirements (at least 50 data points before intervention) and evidence from the prior Lithuanian studies, we selected the two most important alcohol control policies: (i) 1 March 2017 (the first quarter of 2017) and (ii) 1 January 2018 (the first quarter of 2018) [23, 24]. The first policy, implemented on 1 March 2017, aimed at increasing excise taxation (112% for beer, 111% for wine, and 23% for ethyl alcohol) and reducing alcohol affordability [23]. The second policy implemented on 1 January 2018, further increased taxation but also reduced alcohol availability by increasing the legal age for purchasing and consuming alcohol and further restricting off-site alcohol sales [23, 24].

A few initial analytical steps for checking the data were necessary to choose statistical models (Supplementary Material S1). First, dependent variables were checked for normal distribution. Following the Shapiro–Wilk normality tests [25] and QQ plots, we conclude that both the rates of homicide perpetrators and proportions of perpetrators under the influence of alcohol follow a normal distribution (Fig. S1). Second, to check the stationarity of the time series, we run Augmented Dickey–Fuller tests [26]. The tests turned out to be statistically significant (*P* < 0.05) suggesting that that the both series are stationary. Third, the Autocorrelation Function (ACF) and Partial Autocorrelation Function (pACF) plots [27] suggested the presence of autocorrelation in the series for rates of homicide perpetrators (Table S1, Figs S2–S4). This evidence was further confirmed using the Ljung–Box tests [28] (*P* < 0.001). Generalized addictive model (GAM) was selected to investigate the autocorrelation and possible nonlinear relationship of time (smoothed seasonality term). On the basis of these modeling results, we confirmed that for rates of homicide perpetrators, models should include autoregression (AR) and moving average (MA) terms (Table S1). Since outcomes of the initial (baseline) GAM models with the smooth term suggested that it is not statistically significant for the rates of perpetrators under the influence of alcohol, to improve model simplicity and interpretability, smooth term was removed. Thus, the final GAM models for rates of homicide perpetrators are adjusted for (i) AR and MA terms an autocorrelation-moving average correlation structure (corARMA) and (ii) linear trends (quarters). Differently from rates of homicide perpetrators, the ACF and pACF plots of proportions of perpetrators under the influence of alcohol did not show any significant autocorrelation nor nonlinear trend of time. Therefore, as the final model in this case we applied a simple generalized linear model [27]. The residuals of the final models were inspected for normality and stationarity.

Separate models were estimated for assessment of immediate level change and slope change (sustained effect) following each of the two policies. The two variables reflecting immediate level change related to the 2017 and 2018 policies were coded with “0” for all quarters before intervention and “1” for all quarters after intervention. The corresponding two variables reflecting slope change were coded as continuous variables, with “0” for all quarters before intervention and incrementally increasing by one unit with each quarter after intervention. Being under the influence of alcohol at the time the homicide was committed may also depend on other confounding factors such as economic factors such as unemployment and general economic performance or wealth. For example, increase in unemployment and decreasing in GDP are generally associated with increasing violent and homicide mortality [29]. In order to control for these economy-related factors, at least partially, our models also included quarterly series of GDP per capita and unemployment rates from Statistics Lithuania as confounders [15]. All analyses were performed using R version 4.3.2. [30] and modified R scripts from prior ITS analyses on alcohol control policy effects in Lithuania [22, 31].

## Results

**Figure 1.**
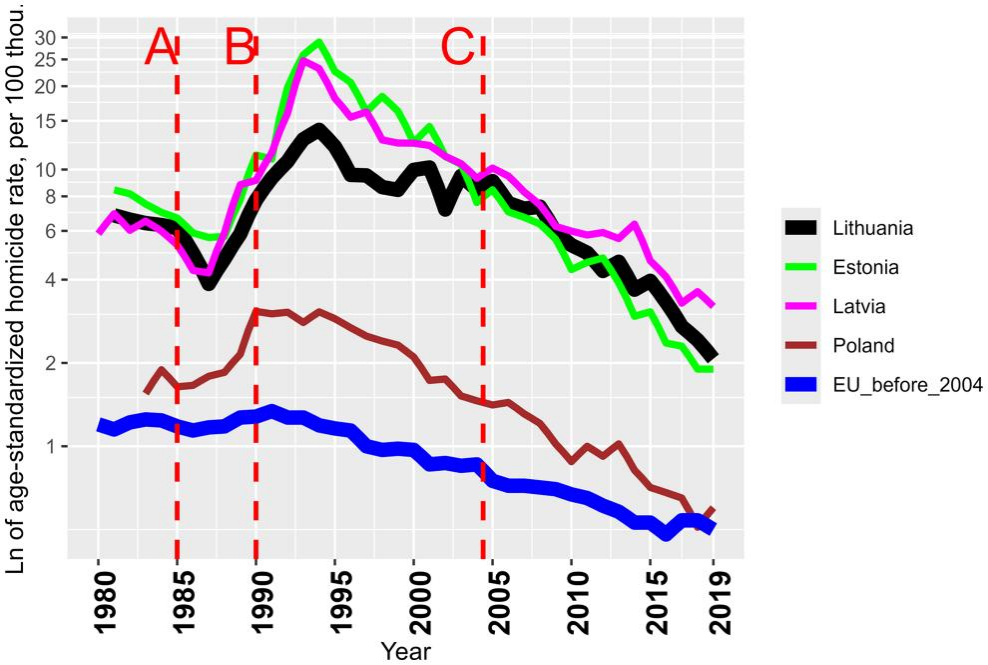
Annual age-standardized homicide death rates (per 100 000 population) in Lithuania, Latvia, Estonia, Poland, and 15 EU (before 2004) countries, 1990–2019 [16, 32].

Figure 1 shows annual trends in age-standardized logarithmized homicide rate in Lithuania, Latvia, Estonia, and Poland, and the average of the 15 EU member countries (EU member states before 2004) from 1981 until 2019. One may observe that higher homicide rates in Lithuania (and the other two Baltic countries) were inherited from the period of Soviet rule. The Lithuanian disadvantage against Poland and EU-15 decreased following 1986 Gorbachev’s anti-alcohol campaign, but it peaked again in the mid-1990s, reaching striking levels of 4–11 times, respectively. Homicide rates in Lithuania started declining again in the second half of the 1990s, but this progress stalled in the first half of the 2000s. This stagnation was responsible for losing an advantage against Latvia and (especially) Estonia, which had, in the beginning 1990s, higher rates than Lithuania. Since 2005, the decline in the homicide rate has been more rapid and systematic, especially from 2015 onwards. Despite this progress, the excess homicide rates in the Baltic countries compared to other EU countries, remain very high. According to the data for 2019, Lithuania’s homicide rate was still 3.5 and 4.1 times higher than in Poland and EU-15, respectively.

**Figure 2.**
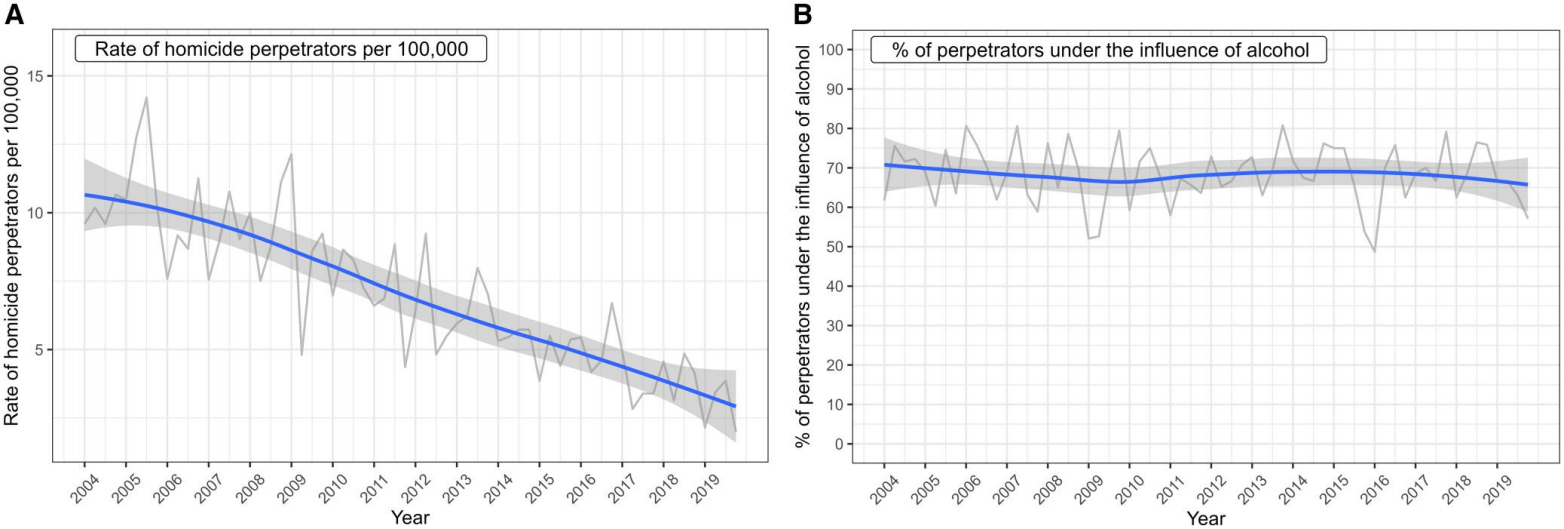
Unsmoothed and smoothed quarterly crude rates of homicide perpetrators (per 100 000 population) (A) and quarterly percentage of perpetrators under the influence of alcohol (B), 2004–2019.

The declining trend can also be observed by exploring crude quarterly rates of homicide perpetrators in Lithuania (Fig. 2). Due to large fluctuations in the observed data, it is difficult to identify the exact magnitude of this decline. The LOESS smoothed homicide rates suggest a more than four times decrease in these rates during the period 2004–19. In 2004, the quarterly crime rates fluctuated between 9.6 and 10.7 per 100 000, whereas they showed much lower values (between 2.0 and 3.9) in 2019. The yearly absolute numbers also declined substantially from 338 perpetrators in 2004 to a low of 80 perpetrators in 2019. Contrary to the progress in declining rates of homicide perpetrators, there were no visible changes in the proportion (percentage) of perpetrators under the influence of alcohol (Fig. 2). These proportions (percentages) remained remarkably high (63%–73%) throughout the entire period.

Model fit statistics provided in Table 1 suggest a better fit (adjusted R2 terms equal to 0.73) for the models for rates of homicide perpetrators, whereas the models for proportions of perpetrators under the influence of alcohol returned very low R^2^ values. Finally, the Log-likelihood tests show that including two socioeconomic control variables does not improve initial models.

**Table 1.** Effects of the 2017 and 2018 alcohol control policies on (A) rates of homicide perpetrators and (B) proportions of perpetrators under the influence of alcohol. Lithuania, 2004–19

	Unadjusted models	Adjusted^a^ models
	Estimated effects and *P*-values	Estimated effects and *P*-values
a) Rate of homicide perpetrators		
2017 Policy		
Level change	−0.371 (*P* = 0.703)	−0.487 (*P* = 0.634)
Sustained effect	0.027 (*P* = 0.827)	0.015 (*P* = 0.912)
AIC	234.18	237.75
BIC	244.97	252.87
Log Likelihood	−112.09	−111.88 (LR test: *P* = 0.808)
Adjusted R^2^	0.733	0.726
2018 Policy		
Level change	0.716 (*P* = 0.547)	0.639 (*P* = 0.603)
Sustained effect	−0.114 (*P* = 0.610)	−0.124 (*P* = 0.598)
AIC	233.96	237.79
BIC	244.75	252.90
Log Likelihood	−111.98	−111.90 (LR test: *P* = 0.919)
Adjusted R^2^	0.734	0.726
b) Proportion of perpetrators under the influence of alcohol		
2017 Policy		
Level change	0.048 (p = 0.310)	0.048 (p = 0.341)
Sustained effect	−0.007 (p = 0.255)	−0.007 (p = 0.312)
AIC	−146.97	−143.81
BIC	−138.33	−130.85
Log Likelihood	77.486	77.903 (LR test: p = 0.659)
Adjusted R^2^	0.021	0.034
2018 Policy		
Level change	0.041 (p = 0.487)	0.033 (p = 0.594)
Sustained effect	−0.012 (p = 0.290)	−0.012 (p = 0.312)
AIC	−147.04	−143.90
BIC	−138.40	−130.94
Log Likelihood	77.519	77.947 (LR test: p = 0.651)
Adjusted R^2^	0.022	0.035

a Additionally controlling for unemployment and GDP.

None of the 2017 and 2018 policy effects reflecting immediate level change or sustained effects as reflected by slope changes turned out to be statistically significant. Only the 2017 policy seems to be at least producing the immediate level change in rates of homicide perpetrators in an expected direction (decrease). Including GDP and unemployment makes the level change more pronounced but still statistically insignificant. The 2018 policy seems to show the opposite result, suggesting the increasing tendency in the rates of homicide perpetrators (Table 1). Meanwhile, in the case of proportions of perpetrators under the influence of alcohol, the level and slope effects were very small.

## Discussion

This study utilized police data on all known homicide perpetrators in Lithuania, and provides the first evidence about the changes in quarterly rates of homicide perpetrators and corresponding proportions of perpetrators under the influence of alcohol. In particular, we aimed to explore the potential role of two different alcohol control policy implementations (one in 2017 and another in 2018). The findings suggest that the continuous decline in homicide rates, rates of homicide perpetrators, and absolute homicide numbers between 2004 and 2019 were not accompanied even by a moderate decline in the proportions of perpetrators under the influence of alcohol.

Between 2004 and 2019, homicide rates declined almost four times from 8.7 to 2.2 homicides per 100 000 population. Meanwhile, despite numerous alcohol control policies implemented since 2008, the proportions of perpetrators under the influence of alcohol remained high during some quarters, sometimes exceeding 70%. Thus, although at a smaller absolute level, homicides likely continued to be mainly committed in heavy alcohol consumption settings, often involving both perpetrators and victims. This assumption stems from other prior studies indicating exceptionally high percentages of victims of violence being under the heavy influence of alcohol [17, 20, 33]. This pattern inherited from the Soviet past seems to prevail in Lithuania [20].

Interrupted time series analyses did not show any significant effects of the two different (combating affordability and availability) alcohol control policies on both the rates of homicide perpetrators and proportions of perpetrators under the influence of alcohol. This finding is quite different from a prior study showing the important impacts of alcohol control policies on reducing alcohol-related trafc harm in Lithuania, including proportions of alcohol-related collisions and crashes, injuries, and deaths [21]. Finally, another study on suicides in Lithuania also reported a significant effect of the 2017 alcohol control policy in reducing suicide mortality, among males in particular [22].

Different from traffic accidents and suicides, homicides in Lithuania seem to be driven more by health and social selection effects [20, 34]. Prior evidence suggests that homicide victims and perpetrators seem to be mainly concentrated within increasingly smaller and marginalized population groups, characterized by extremely high alcohol consumption levels [20]. Therefore, our findings tentatively suggest that implementing universal alcohol control measures may be much less effective in reducing both homicide rates and involvement of alcohol among perpetrators because heavy alcohol consumption habits may be driven by many other, often complex, and nuanced contextual and individual factors. Scarce evidence from Lithuania reports important differences in the age and sex of perpetrators in terms of their victims [34]. For example, the study by Dobryninas *et al.* [34] found that younger males (90% of perpetrators) tend to commit homicides in a group, and their victims tend to be predominantly nonrelatives. In contrast, older perpetrators tend to target relatives, including spouses. The same study also highlights specific motives by younger male perpetrators committing crimes in a group—such as striving to be recognized [34]. This aspect of committing homicide crimes may be related to the prevailing masculinity culture in certain disadvantaged male groups [35]. In the contexts of persisting strong stigmas towards mental health and low family and social support, disadvantaged male groups are very prone to various forms of (auto)aggression, such as suicide and homicide [36].

### Limitations

This study has several important limitations which should be considered before interpreting the results. First, the data on perpetrators and homicide crimes exclude a small number of homicide cases with unknown perpetrator. For example, there were only six homicides with unknown perpetrator(s) in 2019. Second, it is unknown whether the perpetrator was ultimately convicted by court for committing homicide. Third, establishing the involvement of alcohol may not always be precise, especially in cases when a perpetrator gets arrested after some time. Therefore, it is possible that the proportion of homicides wherein the perpetrator was under the influence of alcohol at the time of the incident may be underestimated. Fourth, the original data were classified by month. However, extremely low or zero monthly numbers of perpetrators under the influence of alcohol lead to large fluctuations, poor model fit (as reflected by negative R^2^ values of the GAM models), and excessive noise. Applying aggregated quarterly values led to more stable trends and better model fit, although this approach may have hidden some specific policy-related changes across months within quarters. In addition, both monthly and quarterly approaches produce similar results. We also tested a Poisson distribution, but the goodness of fit and other tests rejected this option. It is possible that applying more complex distributions or statistical models that rely on stronger assumptions may lead to a better fit. However, it is unlikely that such models will change the overall conclusions drawn here. Finally, the time points after the policy interventions may have been too few to show significant effects [37].

## Conclusion

Both a strong reduction of homicide harm and the implementation of strong alcohol control policies in Lithuania were not accompanied by a corresponding decrease in the proportion of homicides wherein the perpetrator was under the influence of alcohol. Despite reductions in alcohol consumption and alcohol-related harm, some very selective segments of Lithuanian society continue to suffer from heavy alcohol consumption and violence. This problem does not seem to able to be addressed by the implementation of alcohol control policies alone and may require more inter-sectorial policy actions. More research is needed to understand homicide determinants from both the victim and perpetrator perspectives. Further studies should explore the associations between alcohol use and homicide in more diverse contexts, also including countries without alcohol policies as controls.

## Supplementary Material

ckaf083_Supplementary_Data

## Data Availability

The original anonymized data on perpetrators comes from the ITCD under the Ministry of the Interior of the Republic of Lithuania. Due to the agreement terms and data protection rules these data cannot be passed to the third party and should be requested directly from the ITCD. The R code used for analyses, will be shared on reasonable request to the corresponding author. Key points There is a lack of evidence about the potential use of alcohol control policies for reducing alcohol-involved homicides in the Central and Eastern European region. The rapid decline in homicide rates in Lithuania was not even accompanied by a moderate decline in the proportion of perpetrators under the influence of alcohol. The results suggest that introducing alcohol control policies alone cannot combat the persistently high impact of alcohol use on fatal violence. In order to develop effective inter-sectorial policies, more research is needed to better understand homicide determinants.
