# Supplementary material for: Persistently high impact of alcohol use on fatal violence in Lithuania despite strengthening alcohol control policies, 2004–19
Source: Eur J Public Health. 2025 Jun 10;35(4):733–7. doi: 10.1093/eurpub/ckaf083 (PMC12311346; doi:10.1093/eurpub/ckaf083)
Supplement: ckaf083_Supplementary_Data [file ckaf083_supplementary_data.pdf]

Supplementary material

Domantas Jasilionis*, Laura Miščikienė, Shannon Lange, Huan Jiang, Daumantas Stumbrys, Olga Meščeriakova, Mindaugas Štelemėkas, Jürgen Rehm

Persistently high impact of alcohol use on fatal violence in Lithuania despite strengthening alcohol control policies, 2004-2019

*Corresponding author: Domantas Jasilionis, Max Planck Institute for Demographic Research, Konrad Zuse Str. 1, 18057 Rostock, Germany, email: jasilionis@demogr.mpg.de

Figure S1. QQ-plots and Shapiro-Wilk normality tests for dependent variables

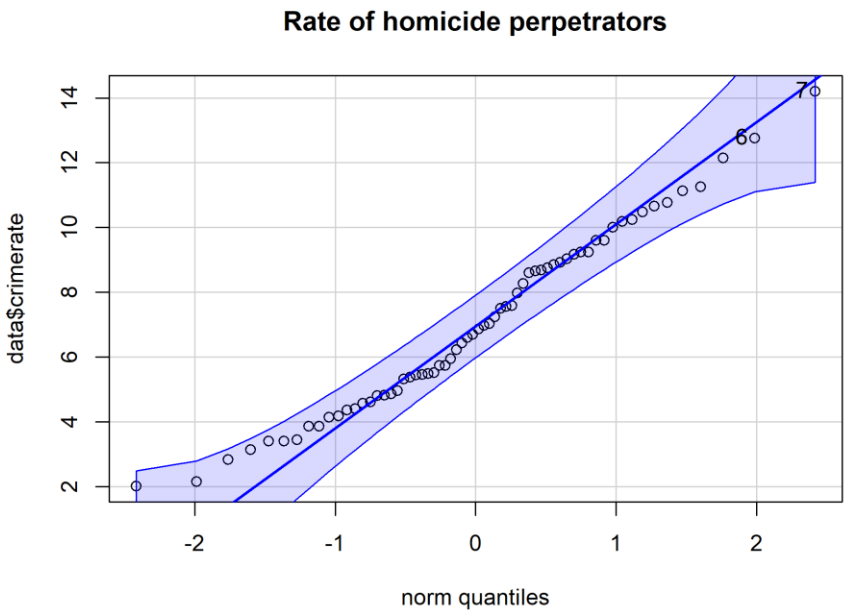

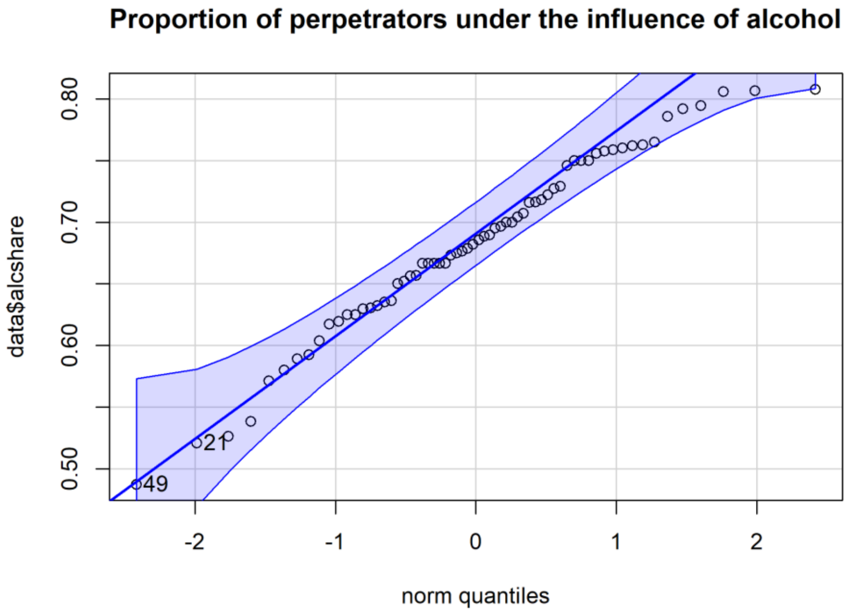

Shapiro-Wilk normality test:

A) Rate of homicide perpetrators: W = 0.97661, p-value = 0.2637

B) Proportion of perpetrators under the influence of alcohol: W = 0.97543, p-value = 0.2301

Table S1. Baseline model checks for stationarity and autocorrelation of time series of a) n=52 quarters between the first quarter of 2004 and the fourth quarter of 2016 and b) n=56 quarters between the first quarter of 2004 and the fourth quarter of 2017.

**Test**	**Rate of homicide perpetrators**		**Proportion of perpetrators under the influence of alcohol**	
	**a) 2017 policy**	**b) 2018 policy**	**a) 2017 policy**	**b) 2018 policy**
**Augmented Dickey-Fuller Test for stationarity**	D-F=-4.80, p-value = 0.01 (stationary)	D-F=-5.10, p-value = 0.01 (stationary)	D-F=-3.89, p-value = 0.02 (stationary)	D-F=-4.12 p-value = 0.01 (stationary)
**auto.arima**	AR=1 & MA=1 required	AR=3 & MA=1 required	no AR & MA required	no AR & MA required
**ACF and PACF plots**	Autocorrelation, AR=1 & MA=1 required	Autocorrelation, AR=1 & MA=1 required	no AR & MA required	no AR & MA required
**Box-Ljung test**	Autocorrelation (p<0.001)	Autocorrelation (p<0.001)	No autocorrelation (p=0.459)	No autocorrelation (p=0.4966)

Figure S2. ACF and pACF plots for Autoarima and Arima (1,0,1) models for rates of homicide perpetrators between the first quarter of 2004 and the fourth quarter of 2016 (before the 2017 policy).

A) Autoarima	Arima (1,0,1)

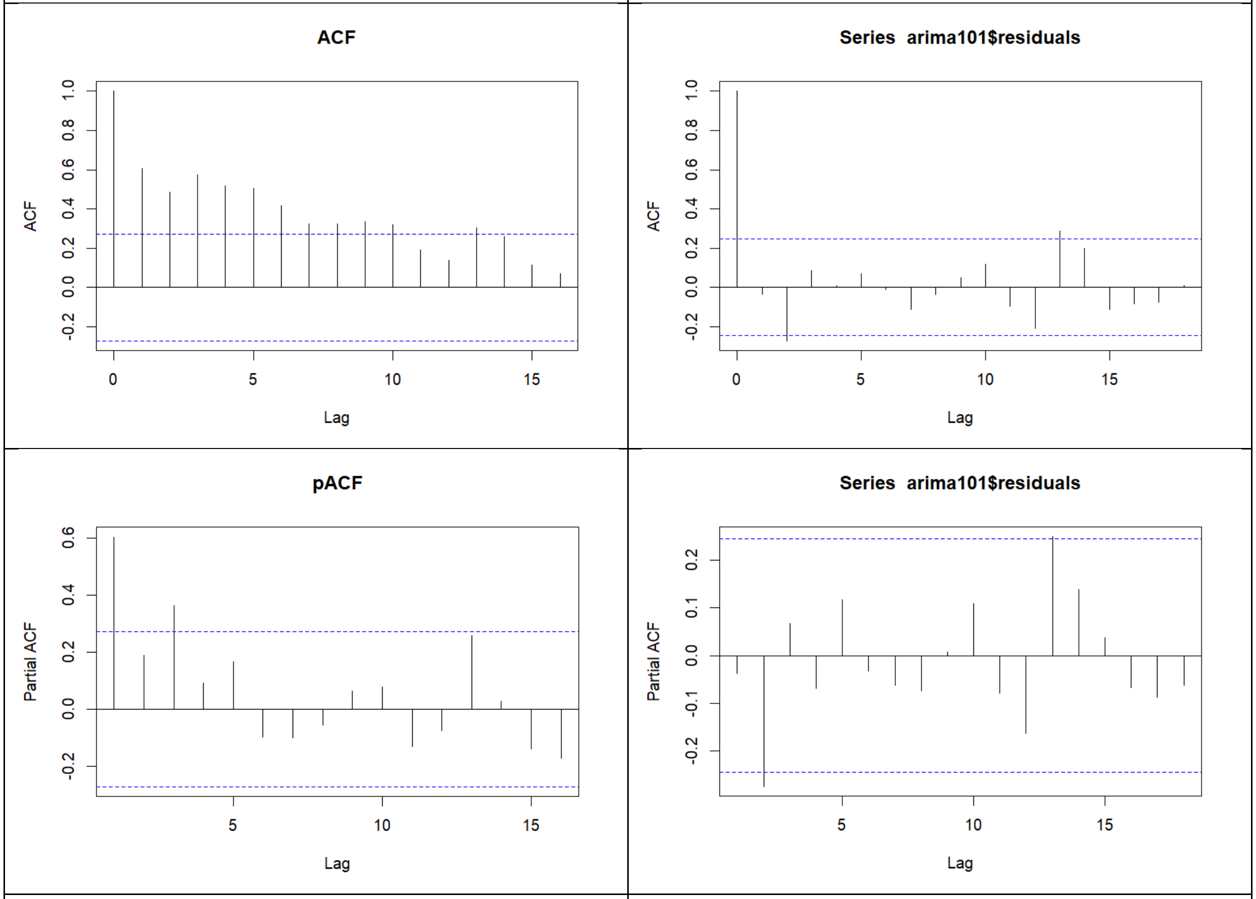

Figure S3. ACF and pACF plots for Autoarima and Arima (3,0,1) baseline models for rates of homicide perpetrators between the first quarter of 2004 and the fourth quarter of 2017 (before the 2018 policy).

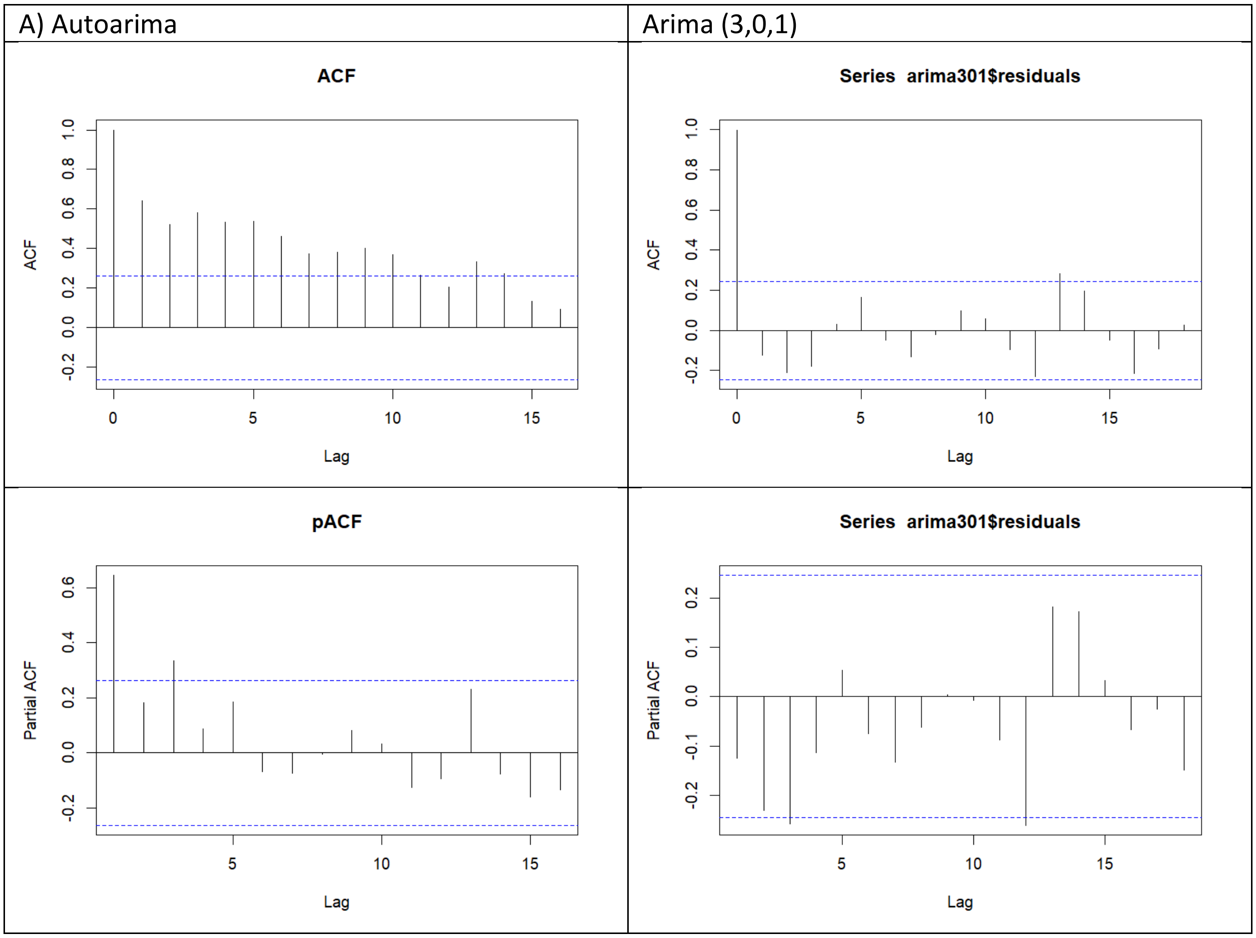

Figure S4. ACF and pACF plots for Autoarima baseline models for proportions of perpetrators under the influence alcohol between a) the first quarter of 2004 and the fourth quarter of 2016 (before the 2017 policy) and b) the first quarter of 2004 and the fourth quarter of 2017 (before the 2018 policy)..

a) Before the 2017 policy

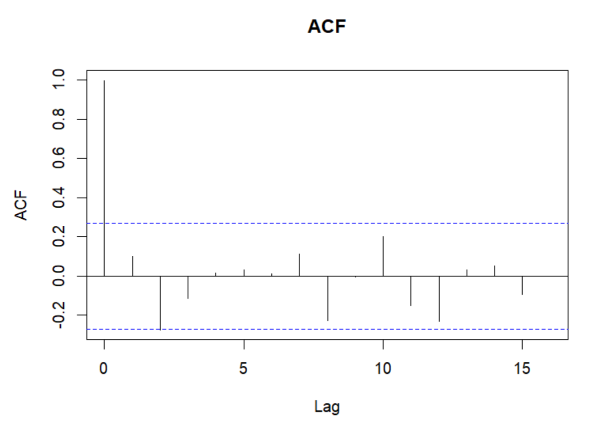

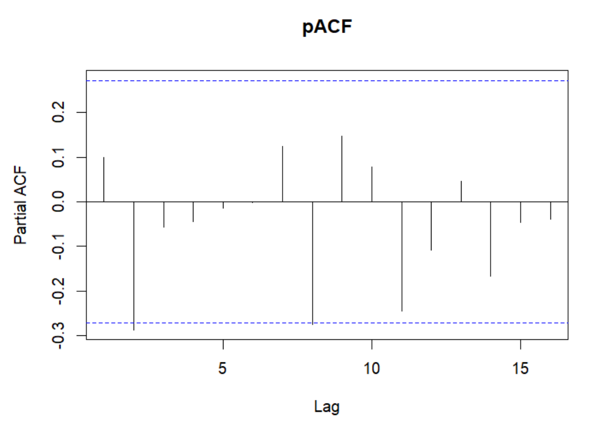

b) Before the 2018 policy

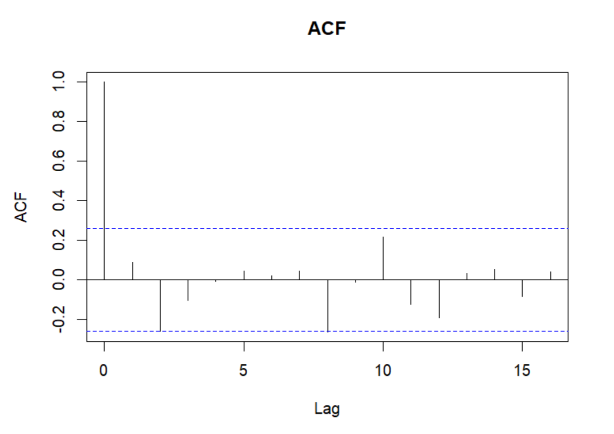

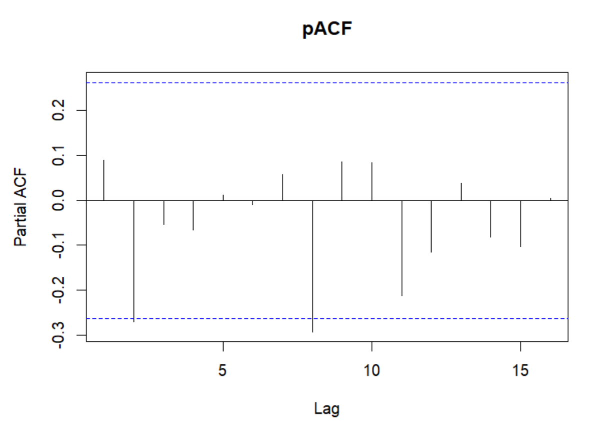

Table S2. Baseline model checks for significance of seasonality and linear trend for time series of a) n=52 quarters between the first quarter of 2004 and the fourth quarter of 2016 and b) n=56 quarters between the first quarter of 2004 and the fourth quarter of 2017.

**Test**	**Rate of homicide perpetrators**		**Proportion of perpetrators under the influence of alcohol**	
	**a) 2017 policy**	**b) 2018 policy**	**a) 2017 policy**	**b) 2018 policy**
a) p-values of smoothed seasonality terms	p=0.895	p=0.914	p=0.513	p=0.662
b) effects of linear trend (quarter) term	**-0.12659** p<0.001	**-0.13111** p<0.001	-0.00046 p=0.515	-0.00016 p= 0.800

Note: a) GAMM models with smoothed seasonality term (cyclic cubic regression splines, 4 knots) and b) GAM models with linear trend (cumulative quarter) term.
